# Innate immune activation profiles are associated with polarization of the T cell response to recombinant arenaviral vectors

**DOI:** 10.3389/fsysb.2026.1897043

**Published:** 2026-09-03

**Authors:** Sarah Szaffich, Valentin Just, Natalija Cvetkovic, Naila Pepic, Michael Schwendinger, Kia Katchar, Klaus K. Orlinger, Henning Lauterbach, Josipa Raguz, Katell Bidet Huang

**Affiliations:** 1 Hookipa Pharma Inc., New York, NY, United States; 2 Medical University of Vienna, Vienna, Vienna, Austria

**Keywords:** immune response, immunotherapy, *in vitro* model, single-cell transcriptomics, viral vector

## Abstract

Arenavirus-based vectors are uniquely positioned to generate durable T cell responses against tumors, which remains a central challenge in cancer immunotherapy. Two members of the arenavirus family, Lymphocytic choriomeningitis virus and Pichinde virus, were genetically engineered to encode a non-oncogenic fusion protein, originating from human papillomavirus 16 E^6^ and E7. Hereafter, these vectors are referred to as HB-201 and HB-202, respectively. HB-201 alone or in combination with HB-202 elicited strong and durable E6/E7-specific CD8+ T cell responses in HPV 16+ cancer patients. Despite their classification within the same virus family, Lymphocytic choriomeningitis virus and Pichinde virus are phylogenetically distant, which may influence virus-host interactions and the pathways leading to antiviral immune responses. Previous studies showed that both vectors infect and activate professional antigen presenting cells. To elaborate on these findings, this study aimed to characterize in depth the innate immune response to HB-201 and HB-202 by single cell sequencing in an in vitro human PBMCs infection model. We confirmed the preferential infection of antigen presenting cells for both vectors, with HB-201 infecting the cross-priming conventional dendritic cell subset cDC1 more efficiently than HB-202. Furthermore, HB-201 induced stronger type I interferon responses than HB-202, as well as quantitative and qualitative differences in inflammatory response associated with a distinct reshaping of monocyte subsets towards intermediate and non-classical monocytes. Antigen presentation and T cell priming cytokines in dendritic cells appeared to favor antiviral CD8+ T cell responses upon HB-201 infection, whereas a more balanced CD4+ and CD8+ priming profile was observed upon HB-202 infection. Immunogenicity data from patients treated with HB-201 and HB-202 confirmed the in vitro findings with functional differences in early T cell response. Thus, despite their shared origins within the arenavirus family, HB- 201 and HB-202 demonstrate distinct differences in early host-virus interaction, potentially resulting in shaping unique T cell responses.

## 1 Introduction

Lymphocytic choriomeningitis virus (LCMV) is the prototypic member of the Arenaviridae family and has been studied for decades as a model virus inducing strong antigen-specific CD8^+^ T cell responses. Arenaviruses are bisegmented enveloped RNA viruses encoding for only 4 viral proteins: the nucleoprotein (NP), glycoprotein precursor (GPC, cleaved into GP1 and GP2), RNA-dependent RNA polymerase (L), and matrix protein (Z). Arenaviruses have low seroprevalence in humans and a high density of N-glycans on their surface proteins making them particularly resistant to neutralisation by antibodies (Sommerstein et al., 2015). Combined with their strong immunogenicity, these characteristics make them attractive vectors for immunotherapy.

To this end, attenuated replication-competent recombinant LCMV vectors carrying transgenic virus- or tumor-associated antigens were created by genetic engineering (Kaller et al., 2017). These constructs elicited high levels of CD8^+^ T cell responses against the encoded antigens and low levels of anti-vector neutralizing antibodies in mouse models. Nonetheless, CD8^+^ T cells directed towards immunodominant LCMV backbone epitopes were found to limit the magnitude of CD8^+^ T cell responses to the encoded antigens after repeated administrations (Bonilla et al., 2021). To further focus immune responses towards transgenic epitopes, a two-vector therapy strategy was established. Pichinde virus (PICV), another member of the arenavirus genus, was modified following the same strategy to carry the encoded antigens. Using recombinant LCMV and PICV vectors in a heterologous alternating immunization regimen resulted in higher levels of transgene-specific CD8^+^ T cell responses and higher therapeutic efficacy in animal models (Raguz et al., 2024; Lauterbach et al., 2021; Katchar et al., 2021; Ho et al., 2023; Edwards et al., 2022).

The immunogenic capacity of viral vectors is thought to be linked primarily to the innate immune stimulation occurring during the virus/host interaction. Upon infection of target cells, the host’s pattern recognition receptors detect molecular patterns associated with virus components or intermediates of replication. This activates type I interferon (IFN) and inflammatory responses, which signals to infected and neighboring cells to mount an antiviral state, preventing the spread of infection (Thompson et al., 2011; Takeuchi and Akira, 2010). The cytokines and chemokines produced act to attract, activate and promote the differentiation of different types of lymphocytes and professional antigen-presenting cells (APCs). APCs process and present viral antigens upon phagocytosis, endocytosis or receptor-mediated uptake, or through direct infection. Activated APCs then migrate to secondary lymphoid organs to orchestrate expansion and differentiation of antigen-specific T and B cells, thereby enabling effector functions and long-term control of the infection (Wan et al., 2024).

The APC subset, the mode of antigen acquisition, and the cytokine environment are critical in shaping subsequent T cell responses. In case of direct infection, conventional type 1 dendritic cells (cDC1s) and conventional type 2 dendritic cells (cDC2s) can present antigens on their Major Histocompatibility Complex (MHC) class I and II molecules and stimulate CD4^+^ and CD8^+^ T cell responses, respectively. In case of exogenously acquired antigens, only cDC1s efficiently cross-present antigens to CD8^+^ T cells and promote strong cytotoxic T-lymphocyte responses, whereas cDC2s preferentially induce CD4^+^ T helper cell differentiation (Theisen and Murphy, 2017; Collin and Bigley, 2018; Tatsumi et al., 2025). Plasmacytoid DCs (pDCs) and monocytes are generally less effective at presenting antigens and priming T cells, but participate in conditioning the cytokine environment through production of IFNs and pro-inflammatory cytokines (Fitzgerald-Bocarsly et al., 2008; Austermann et al., 2022). Viral infections and intracellular bacteria generally induce type I IFN and IL-12 expression, which promote the differentiation of Th1-polarized CD4^+^ T cells and cytotoxic CD8^+^ T cells, whereas extracellular bacteria and fungi induce IL-17-mediated responses through Th17-polarized CD4^+^ T cells, and parasites predominantly induce IL-4, which promotes Th2-polarized CD4^+^ T cell responses, although considerable flexibility and overlap exist between these pathways (Saravia et al., 2019).

Because viruses and the immune system have evolved together for millions of years, viruses have developed countermeasures to avoid detection or actively block antiviral defenses (Loo and Gale, 2007; Zhu et al., 2023). While the same pathways are generally targeted, such as suppression of IFN responses or antigen presentation, the exact mechanisms can vary even between closely related viruses. For instance, SARS-CoV-2 uses viral proteins Nsp1 and Orf6 to strongly suppress IFN signaling and host translation, resulting in a delayed antiviral response and T cell priming (Shemesh et al., 2021; Lei et al., 2020). Conversely, SARS-CoV-1 induces a more rapid and pronounced inflammatory response due to differences in Orf3b and Orf6 activity. Herpesviruses HSV-1 and HSV-2 differ in how they interfere with MHC-I antigen presentation: HSV-2’s ICP47 protein blocks TAP-mediated peptide loading more efficiently than HSV-1, resulting in reduced CD8^+^ T-cell recognition at the site of infection, whereas HSV-1’s comparatively weaker TAP inhibition permits stronger local CD8^+^ T-cell activation but triggers greater inflammation in neuronal tissues (Zheng et al., 2012; Pan et al., 2025). These examples illustrate how even minimal divergence between related viruses can reconfigure the quality, magnitude, and durability of T-cell responses.

Although both LCMV and PICV belong to the same Arenaviridae family, they belong to distinct phylogenetic clades with different natural histories. LCMV is a globally distributed Old World arenavirus, which infects the common house mouse (*Mus musculus)*. PICV belongs to the New World phylogenetic lineage and is primarily found in Colombia, infecting rice rats (*Oryzomys albigularis*) with minimal known human exposure (Buchmeie et al., 1974; McLay et al., 2014). Thus, despite sharing genomic architecture and a high degree of homology, it is likely that these two viruses show notable differences in their interactions with the human immune system. This could in turn shape adaptative responses to immunotherapy when used as viral vectors.

In this study, we aimed to characterize and compare the human immune response to LCMV and PICV viral vectors. We developed a human PBMC *in vitro* infection model to characterize the response to the LCMV- and PICV-based vectors HB-201 and HB-202, respectively, both encoding the same HPV-16 E^6^ and E7 fusion protein (Schmidt et al., 2020). Using scRNAseq, we showed that while both vectors preferentially infected APCs, differences in tropism could be observed for cDC subsets. We also identified fundamental differences in IFN and inflammatory responses to the two vectors, with the potential to explain differential polarization of the T cell responses.

HB-201 and HB-202 were tested in a phase I/II clinical trial for the treatment of HPV-16^+^ head and neck squamous cell carcinoma (HNSCC) (H-200-001, NCT04180215). In the phase I part of this study, patients with metastatic/recurrent disease were treated with HB-201 alone or HB-202/HB-201 alternating two-vector therapy, in a dose-escalation study. Exploratory analyses performed included measurement of blood cytokine responses to evaluate innate immune responses to treatment, and of T cell responses to the HPV E7E6 transgene were measured at baseline and after treatment. Using this exploratory immunogenicity data, we confirmed that priming with HB-201 or HB-202 could generate T cell responses with different functional properties.

## 2 Results

### 2.1 Establishment of a human PBMC *in vitro* infection system

**FIGURE 1:**
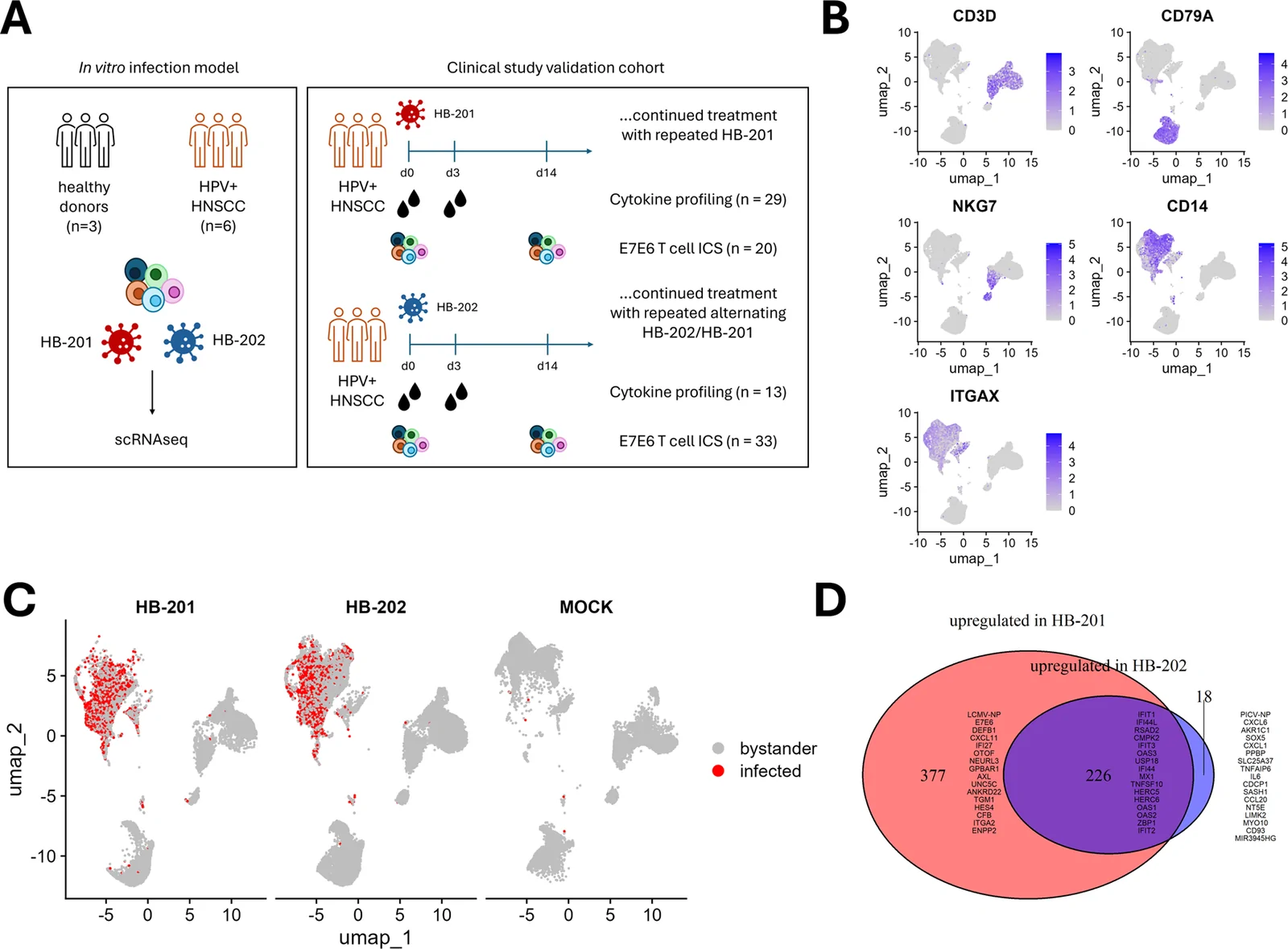
Set-up of *in vitro* PBMC infection system. **(A)** Experimental design. **(B–D)** PBMCs from 9 donors were mock-infected or infected with HB-201 or HB-202, enriched for HLA-DR-expressing cells and processed for scRNAseq. **(B)** UMAP with expression of main cell type markers CD3D (T cells), CD79A (B cells), NKG7 (NK cells), CD14 and ITGAX (monocytes and DCs). **(C)** Detection of infected cells via expression of viral RNA (in red). **(D)** Venn diagram of upregulated genes in HB-201 or HB-202 infection condition vs. MOCK.

Previous studies have shown that arenavirus preferentially infect APCs *in vitro* and in animal models (Raguz et al., 2024). To characterize the innate immune responses to HB-201 and HB-202 infection in the human host, we opted for human PBMCs, which naturally contain different subsets of APCs: cDC1, cDC2, plasmacytoid DCs (pDCs) and monocytes. HB-201 and HB-202 are in development to treat patients with HPV-16+ HNSCC, and it is known that patients with cancers at an advanced stage often present with a dysfunctional immune system. Therefore, to be representative of the target population, we selected PBMCs from three healthy donors and six HPV-16^+^ HNSCC patients from the Phase I H-200-001 study obtained at baseline before the start of treatment. We analyzed these PBMCs after infection with HB-201, HB-202 or mock infection by scRNAseq, to provide granular information about virus tropism and innate immune activation at the cell level and uncover any differences in responses even if those were restricted to rare cell subsets (Figure 1A).

We also obtained data from H-200-001 Phase I patients treated with HB-201 monotherapy or HB-202/HB-201 alternating two-vector therapy for clinical validation of the findings (Figure 1A). Cytokine profiling at baseline and 3 days after treatment, representing the early innate immune response to the viral vector, was available for 42 patients (29 having received one dose of HB-201, 13 having received one dose of HB-202). E6 and E7-specific intracellular cytokine staining results of T cells were available at baseline and 14 days after treatment for 53 patients (20 having received one dose of HB-201, 33 having received one dose of HB-202) to quantify the magnitude and functional profile of the transgene-specific T cell response.

Because arenavirus genomic RNA is not polyadenylated, we hypothesized that virus-specific primers may be required for the detection of viral RNA in infected cells. In order to optimize viral RNA detection, we tested different virus-specific primers using Nanopore long-read sequencing. Surprisingly, but as described previously for other non-polyadenylated viruses (Sanborn et al., 2020), sequencing of LCMV and PICV genomes was as efficient with oligodT as with different viral 3′ specific primers (Supplementary Figure S1). Therefore, we used the standard oligodT primers with the 5′ immune profiling Kit (10x Genomics) for scRNAseq, without protocol modification. Sequences of the viral open reading frames (NP, GP, L, Z and E7E6) for HB-201 (LCMV) and HB-202 (PICV) were appended to the human reference genome, generating an extended reference to map virus-derived reads.

We optimized the PBMC infection conditions using GFP reporter vectors. Infection at a multiplicity of infection (MOI) of 1 for 24 h provided the best balance between transgene expression and cell viability for both vectors and was used for all subsequent experiments (Supplementary Figure S2). Each PBMC sample was infected either with HB-201, HB-202, or mock infected. Because PBMCs contain less than 20% of APCs, we enriched for APCs before scRNAseq by HLA-DR positive selection using magnetic beads to increase the proportion of APCs in the sequencing data. Flow cytometry analysis of the PBMC fractions before and after enrichment showed that HLA-DR enrichment increased the proportion of HLA-DR + cells to over 80% and that cell depletion was limited to CD3^+^ and CD3^−^ CD19^−^cells (Supplementary Figure S3). All APC subtypes including monocytes, cDC1, cDC2 and pDC were represented after enrichment, suggesting that this step did not introduce unintended bias for subsequent scRNAseq.

Sequencing quality control metrics (Supplementary Figure S4A) before filtering indicated a median of 2,829 sequenced cells per sample (2,104 min and 3,807 max), with a median of 7,620 detected transcripts (488 min and 467,127 max) mapped to 2851 genes per cell (199 min and 12,885 max) and a low proportion of mitochondrial RNA reads (median of 3.33%, 0.00% min and 88.53% max). The parameters during the filtering process were configured to exclude cells with fewer than 100 detected genes, eliminating empty droplets and debris. Cells with more than 8,000 detected genes or more than 50,000 unique molecular identifiers (UMIs) were excluded to minimize the inclusion of multiplets. Cells with greater than 15% mitochondrial gene expression were excluded to remove stressed and apoptotic cells. After applying these filtering steps, a median of 89.07% cells per sample were retained (76.85% min and 96.77% max). A cell was defined as infected if viral RNA of one of the four arenaviral coding regions **(**NP, GP, L, Z**)** or E7E6 was detected. Viral RNA of the respective vector backbone was detected in all infected samples, whereas no infected cells were detected in the mock-infected condition. The overall number of infected cells ranged from 5.76% to 44.43% (median = 24.40%) for HB-201 and from 4.62% to 28.94% (median = 16.30%) for HB-202 across all donors (Supplementary Figure S4B).

Uniform manifold approximation and projection (UMAP) was computed from the scRNA-seq data to resolve the major cellular clusters. HB-201 and HB-202 viral RNAs were detected almost exclusively in the UMAP region characterized by expression of *CD14* and *ITGAX*, two markers of monocytes and DCs, which is consistent with the previously described infection pattern of LCMV and PICV (Figures 1B,C) (Raguz et al., 2024). Pseudobulk differential gene expression analysis for HB-201- and HB-202-infected samples vs. mock-infected samples showed that HB-201 and HB-202 both upregulated a shared set of 371 genes (Figure 1D). Many of these genes could be mapped to the type I IFN pathway (*IFIT1*, *IFI44L*, *IFIT3*, *OAS3* or *MX1*), consistent with the activation of an antiviral immune response. In addition, HB-201 and HB-202 upregulated 543 and 88 unique genes, respectively, suggesting major differences in the virus recognition pathways triggered by the two vectors.

Together, these preliminary analyses suggested that our *in vitro* PBMC infection model reproduced key features of arenavirus infections and is a useful tool to characterize the innate immune responses to HB-201 and HB-202.

### 2.2 HB-201 infects cDC1 more efficiently than HB-202

**FIGURE 2:**
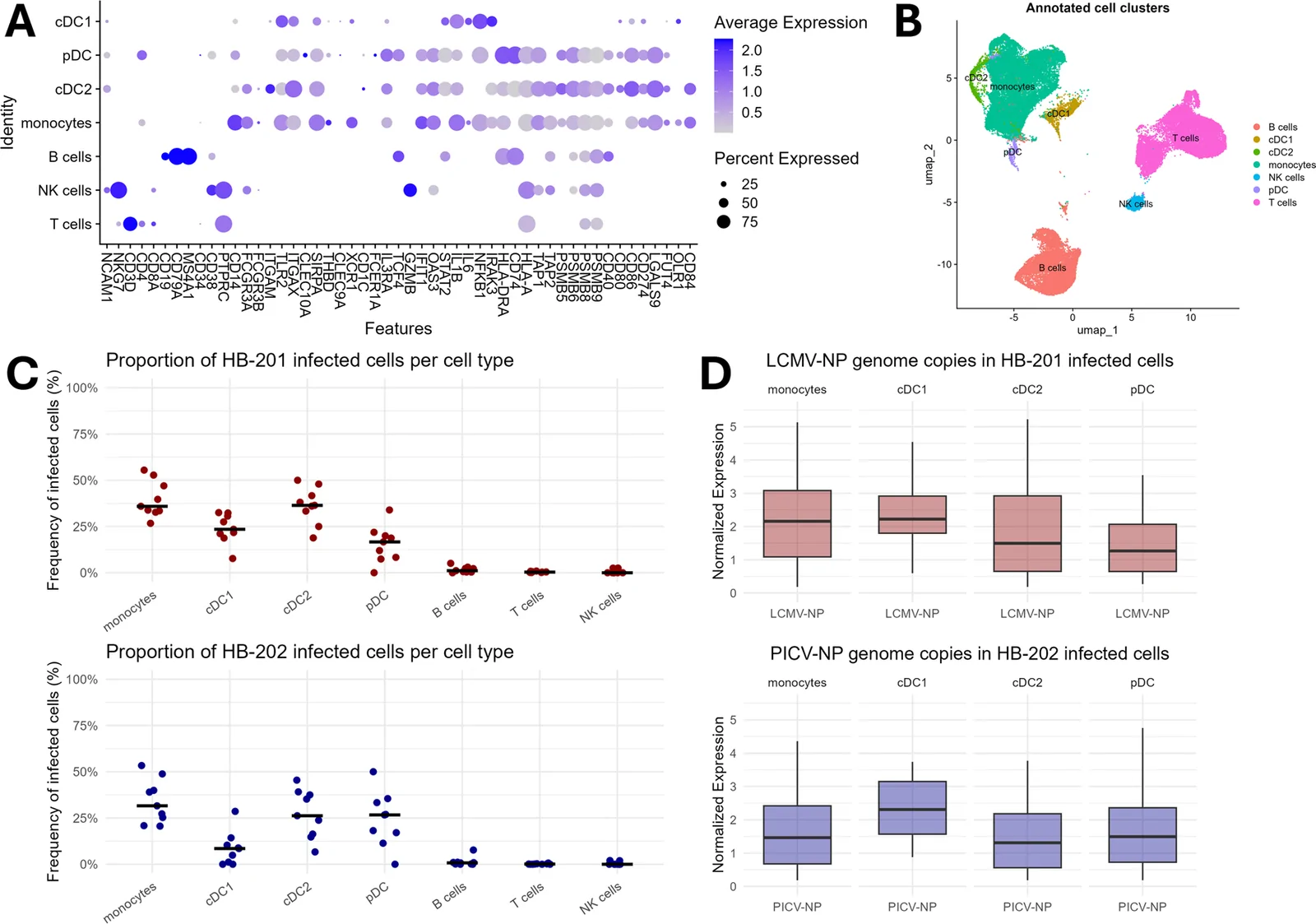
HB-201 and HB-202 selectively infect antigen presenting cells. **(A)** Manual cell type annotation based on a selected set of genes. **(B)** UMAP plot of annotated cell types. **(C)** Proportion of infected cells in each annotated cell type. Horizontal lines indicate the median proportion per cell type. **(D)** Viral NP genome copies measured by normalized gene expression in infected cell types. Box plots display the median (center line), interquartile range (box), and whiskers extending to the most extreme values within 1.5× the interquartile range.

Unsupervised clustering using the Leiden algorithm was applied to identify transcriptionally distinct populations. The *clustree* (Zappia and Oshlack, 2018) package was used to visualize how cell clusters changed across different clustering resolutions (Supplementary Figure S5). As the objective of the experiment was to examine the response of APC subsets to HB-201 and HB-202 infection, it was necessary to obtain sufficient granularity to identify distinct subsets which could be attributed to cDC1, cDC2, pDC and monocytes. No clustering resolution identified each subset as a single cluster, likely due to the large differences in relative proportion of the cell types and to the effect of viral infection on the cell’s transcriptomes. Therefore, we selected the resolution of 1.2, identifying 32 clusters, and manually merged clusters to obtain an annotation reflecting known signatures of each PBMC subset (Collin and Bigley, 2018; Heger et al., 2023) (Figure 2A). As we could not identify standard signatures of DC subsets in the context of PBMCs, the annotated subsets may not match the classical flow cytometry-based definition but can nonetheless enable comparison between HB-201 and HB-202. T cells were defined by the expression of *CD3D*, B cells by *CD19*, *CD79A* and *MS4A1*, and NK cells by *NCAM1* and *NKG7* expression. To differentiate APC subsets, clusters characterized by expression of *CD14* and *FCGR3A* were identified as monocytes. DC subsets were defined as cells with lower expression of *CD14* and expression of a combination of markers: *FCER1A*, *IL3RA* and *TCF4* for pDCs, *XCR1* and *CLEC9A* for cDC1 and *CD1C* for cDC2. 6 clusters expressing marker combinations not corresponding to any classical PBMC cell type were excluded.

In total, 66,938 cells were annotated (Figure 2B). These included 13,039 B cells (median per sample = 495, min-max = 38-1,021); 18,771 T cells (median per sample = 734, min-max = 220-1,594); 1,608 NK cells (median per sample = 59, min-max = 3-141); 29,270 monocytes, (median per sample = 1,066, min-max = 468-2,161); 2,024 cDC1 (median per sample = 37, min-max = 10-438); 1,513 cDC2 (median per sample = 39, min-max = 4-182) and 713 pDC (median per sample = 22, min-max = 6-56).

As previously observed, both HB-201 and HB-202 selectively infected APCs (Figure 2C) (Raguz et al., 2024). The median infection rate for B cells was 1.13% and 0.75%, for T cells 0.35% and 0.10%, and for NK cells 0% and 0% for HB-201 and HB-202 infection, respectively. In contrast, viral RNA was detected in a median of 35.92% and 31.58% of monocytes, 23.49% and 8.49% of cDC1, 36.43% and 26.23% of cDC2, and 16.67% and 26.67% of pDCs for HB-201 and HB-202 respectively. Thus, all APC subsets were infected by both HB-201 and HB-202. Monocytes showed the highest overall infection rates (median HB-201 = 35.92%, median HB-202 = 31.58%), followed by cDC2 (median HB-201 = 36.43%, median HB-202 = 26.23%) and pDCs (median HB-201 = 16.67%, median HB-202 = 26.67%). cDC1 exhibited notable differences in infectability between the two vectors, with between 7.69% and 32.5% cells infected by HB-201 (median = 23.49%) and between 0% and 28.6% cells infected by HB-202 (median = 8.49%). Although the tropism of the two vectors was generally similar, we noted a marked reduction in the infection efficiency of cDC1 for HB-202 compared to HB-201. Of note, two donors showed no sign of HB-202 infection in cDC1.

We quantified the levels of LCMV-NP and PICV-NP RNA in infected cells by normalizing raw transcript counts to adjust for differences in sequencing depth across cells. We found the highest viral RNA levels in monocytes and cDC1 infected with HB-201 compared to cDC2 and pDCs. For HB-202, we observed that despite a lower infection rate, infected cDC1 presented the highest viral load, whereas other APC subsets had higher infection rates but lower viral load.

Overall, this analysis confirms that both HB-201 and HB-202 vectors exhibit broad tropism for all APC subsets, although HB-202 shows a comparatively reduced infection efficiency in cDC1.

### 2.3 HB-201 is a stronger activator of type I interferon responses than HB-202

Next, we examined the cell subset-specific innate immune response to HB-201 and HB-202 infection. The type I IFN response is one of the first lines of defense against viruses. Extracellular and intracellular pattern recognition receptors (PRRs) detect molecular patterns associated with viruses and activate IRF3/7- and NF-kB-dependent signaling cascades leading to the production of IFN alpha and beta, which act on the IFN alpha receptor (IFNAR) on the infected cell and bystander cells to induce the production of interferon-stimulated genes (ISGs) with antiviral activities. Different patterns of expression of PRRs can influence type I IFN responses. pDCs, with high expression of *TLR7* and *TLR8*, are considered specialized producers of type I IFNs. There are also several differences in the expression of PRRs between cDC1 and cDC2 (Carroll et al., 2024). However, all APCs and generally all cell types are capable of engaging in IFN responses to control infections. In turn, viruses have evolved different strategies to avoid induction of IFN responses or escape their antiviral effects.

To analyze the activation of type I IFN responses upon HB-201 and HB-202 infection, we defined a type I IFN activation gene module composed of representative members of the pathway: core IFN-I production (*IFNB1*, *IRF7*), canonical receptor/kinase signaling (*IFNAR1/2*, *JAK1*, *TYK2*), transcriptional effectors (*IRF9*, *STAT1* and *STAT2*), IFN-stimulated genes (ISG) effectors (*MX1*, *OAS1*, *ISG15*), and downstream signaling factors (*EGR1*, *CCL7*) (Yang et al., 2023; Ma et al., 2023; Hu et al., 2021; Piehler et al., 2012; Verhelst et al., 2012).

**FIGURE 3:**
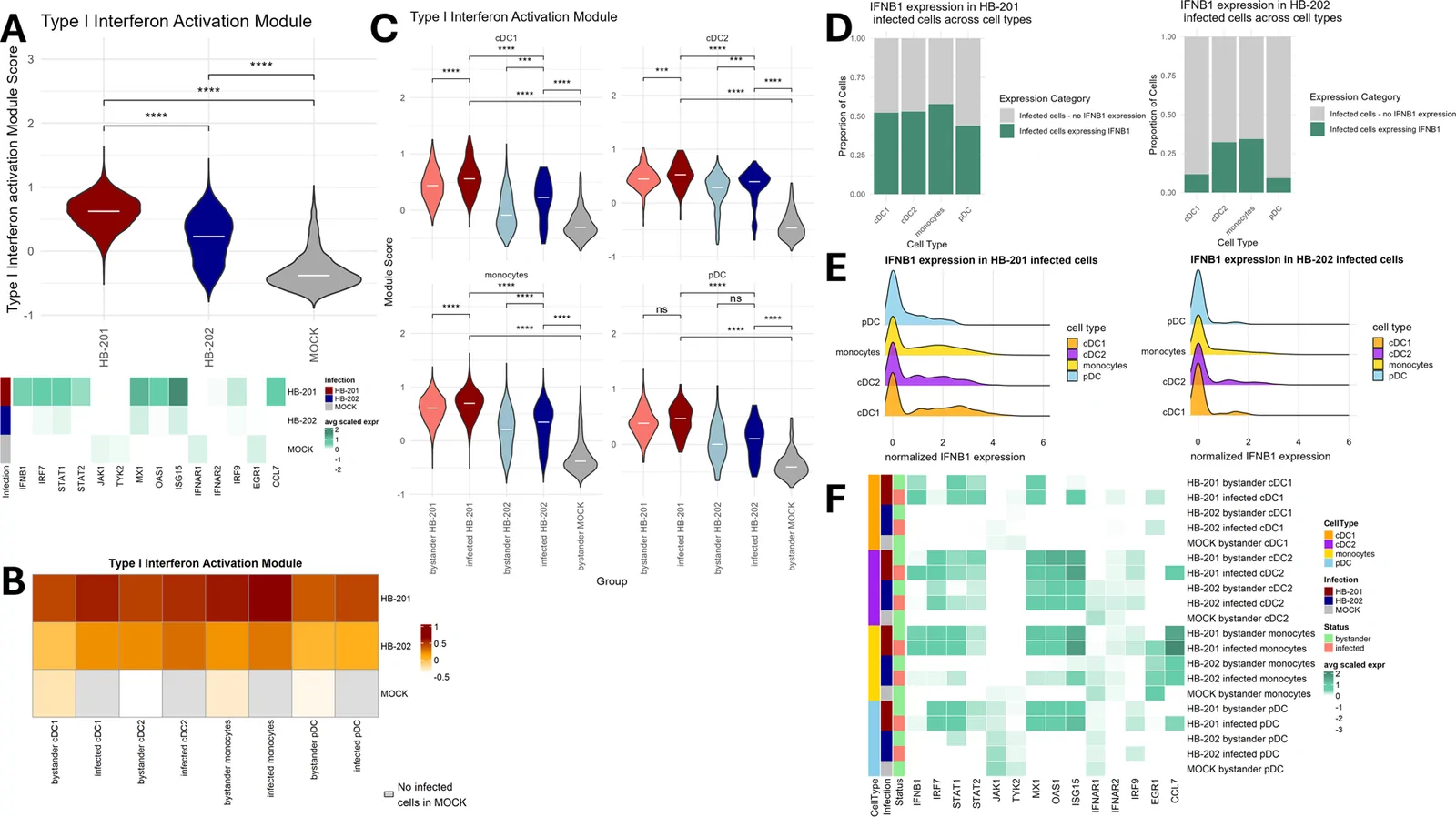
HB-201 is a stronger activator of IFN type I responses than HB-202. **(A)** Overall expression of genes included in module split by infection condition. **(B)** Expression of Type I Interferon activation module in each infection condition across infected and bystander cell types. **(C)** Violin Plots visualizing significant differences between infection conditions within one cell type assessed using Wilcoxon rank-sum test with unadjusted p-values (*p < 0.05, **p < 0.01, ***p < 0.001, ****p < 0.0001). **(D,E)** Proportion and gene expression level of infected cells expressing IFNB1. **(F)** Scaled gene expression of genes included in Type I Interferon activation module split by infection condition, cell type and infection state.

Overall, upregulation of the type I IFN activation module was detected upon both HB-201 and HB-202 infection when compared to mock infection (Figure 3A). Module scores were increased in all APC populations (cDC1, cDC2, monocytes and pDCs) in infected cells. IFN responses were also detected in bystander cells, albeit to a lower level, reflecting the paracrine ability of type I IFN responses to alert neighboring cells and trigger antiviral states (Figure 3B). Overall, HB-201 was a stronger activator of the type I IFN module than HB-202 across all APC subsets (Figure 3C).

When examining the expression levels of *IFNB1* as the driver of the response, we observed that *IFNB1* was induced in all APC subsets upon HB-201 infection, whereas *IFNB1* levels were lower upon HB-202 infection (Figure 3D). The reduced *IFNB1* induction by HB-202 despite comparable infection rates and viral load as for HB-201 (Figure 3E) suggests that PICV has evolved more efficient strategies to either keep viral genomes from being detected and/or actively antagonize the induction of the response. Interestingly, although pDCs are considered specialized type I IFN producers, we did not observe higher *IFNB1* expression in our pDC cluster compared to other clusters for either HB-201 or HB-202. This may be due to contamination by a different cell type in this cluster diluting the signal, or because both viruses have evolved pDC-specific mechanisms of antagonizing type I IFN production.

Generally, the induction of *IFNB1* was accompanied by a proportional induction of downstream signaling, represented by *STAT1* and ISGs (Figure 3F). Nonetheless, some cell subset-specific differences and virus-specific differences could be observed. For instance, the ISG *OAS1* was induced in all infected cell subsets but cDC1, whereas increased levels of *MX1* and *ISG15* were measured in all cell subsets without exception. CCL7 was expressed in response to infection by HB-201 and HB-202 in monocytes, but to a much lower extent and only in response to HB-201 in cDC2 and pDCs. *IFNAR1* appeared to be downregulated in response to HB-201 infection, but not in response to HB-202, in all cell subsets, potentially as part of a feedback loop to curb the effects of high IFN responses on the host and avoiding immune pathology.

This data indicates that although human APCs mount type I IFN responses upon HB-201 and HB-202 infection, HB-202 more efficiently evades or antagonizes the induction of *IFNB1*, dampening the induction of downstream antiviral effectors.

### 2.4 HB-201 and HB-202 induce different subsets of pro-inflammatory cytokines

In addition to inducing type I IFN responses, the recognition of viruses by PRRs also triggers inflammatory signaling cascades, such as activation of NFkB. This leads to the production of pro-inflammatory cytokines and chemokines, which attract and activate immune cells, including neutrophils, macrophages and T cells to the site of infection, enabling elimination of infected cells. To characterize the inflammatory response to HB-201 and HB-202 infections, we defined an Inflammation gene module composed of representative pro-inflammatory mediators and their regulators. This module included genes encoding for cytokines (*TNF*, *IL1A*, *IL1B*, *IL6*, *IL18*, *IL1RN*) and chemokines recruiting monocyte, macrophage and T cells (*CCL2*, *CCL3*, *CCL4*, *CCL5*, *CCL20*) or neutrophils (*CXCL1*, *CXCL2*, *CXCL3*, *CXCL5* and *CXCL8*), genes involved in the assembly and activation of the inflammasome (*NLRP3*, *AIM2*, *NLRC4*, *PYCARD*, *CASP1*, *CASP4*, *GSDMD*), pattern recognition (*TLR3*, *TLR7*, *TLR8*, *NOD2*) and downstream effector functions (*PTGS2*, *S100A8*, *S100A9*).

**FIGURE 4:**
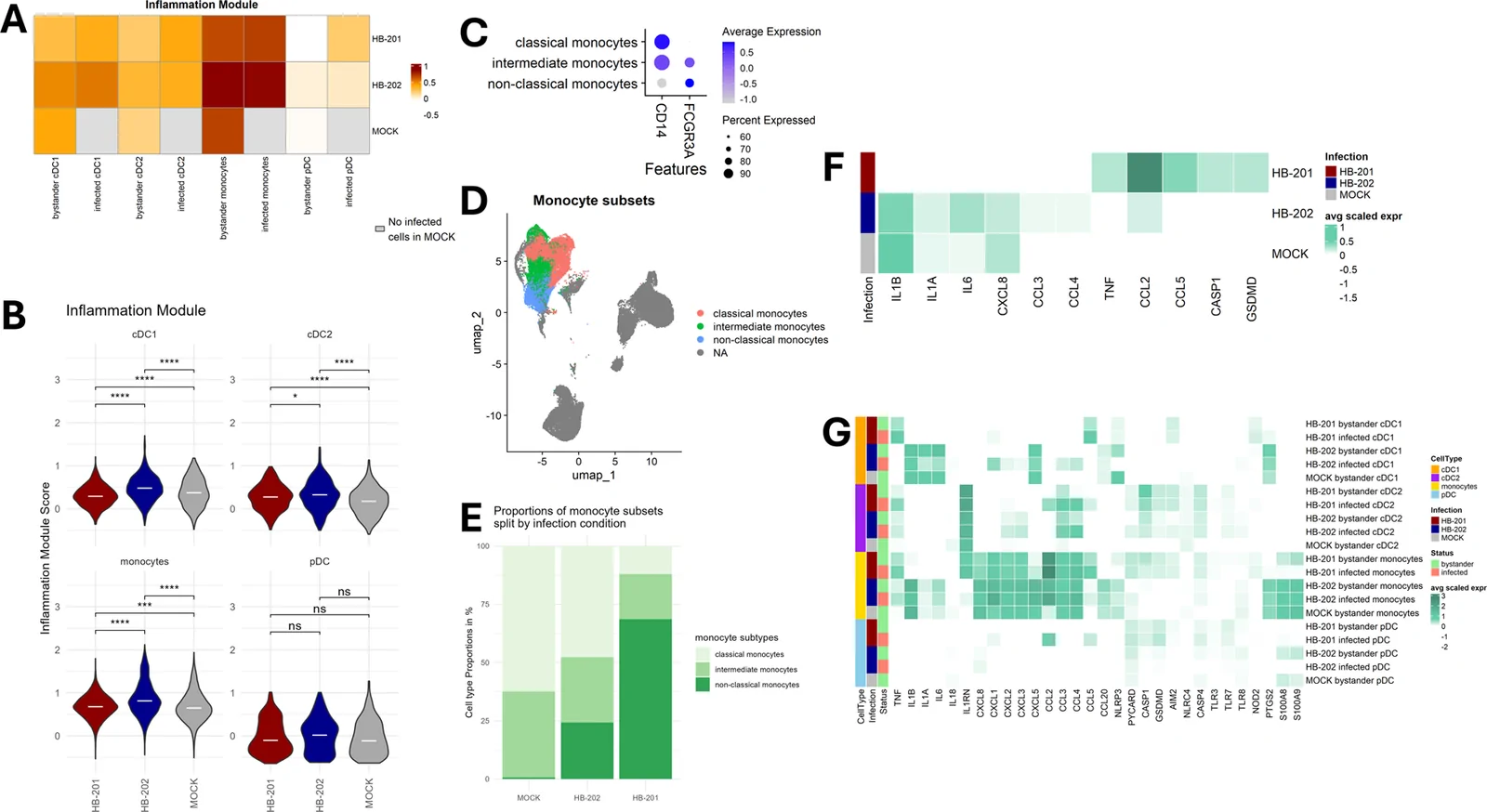
Monocytes as drivers of distinct types of inflammatory responses between HB-201 and HB-202. **(A)** Expression of inflammation module in each infection condition across infected and bystander cell types. **(B)** Significantly different expression of Inflammation Module between infection conditions was assessed using Wilcoxon rank-sum test with unadjusted p-values (*p < 0.05, **p < 0.01, ***p < 0.001, ****p < 0.0001). **(C–E)** Monocyte subsets were identified by expression of CD14 and FCGR3A. Proportion of each subtype within all monocytes was calculated for each infection condition. **(F)** Selected genes from the inflammation module indicate a distinct expression profile between HB-201 and HB-202. **(G)** Scaled gene expression of all inflammation module genes split by cell type, infection condition and status.

Differential module scoring revealed elevated inflammatory activity in all cell types infected by HB-201 and HB-202 except pDCs (Figures 4A,B). In contrast to what was observed for the IFN type I module, HB-202 was a stronger activator of the Inflammation module, when compared to HB-201. Monocytes were clear drivers of the inflammatory response to infection for both viruses, with scores at least 1.87 fold superior to other cell types (Figures 4A,B). When examining the proportion of monocyte subsets in each infection condition, we noted that HB-201 induced a much larger reshaping of the monocyte compartment than HB-202. In mock-infected samples, a median of 79.57% of the cluster (min 3.61% to max 98.74%) was mapped to classical monocytes, defined as the subset expressing CD14 (encoded by *CD14*) but not CD16 (encoded by *FCGR3A* and *FCGR3B*); a median of 20.34% (min 1.26% to max 94.99%) were defined as intermediate monocytes, with high expression of CD14 and CD16; and a median of 0.25% (min 0.09% to max 4.05%) were defined as non-classical monocytes, with reduced CD14 and high CD16 expression (Figures 4C–E). After 24 h of HB-201 infection, a median of 80.89% (min 8.11% to max 97.39%) of the monocytes were non-classical, whereas this value was only a median of 5.96% (min 0.23% to max 97.15%) for HB-202. Infection efficiency was comparable across subsets for both vectors (Supplementary Figure S6).

Classical monocytes are the first responders to infection, producing pro-inflammatory cytokines such as TNF-α, IL-6, IL-1β upon PRR stimulation. They can differentiate into intermediate monocytes, which exhibit higher antigen presentation capacity and produce more reactive oxygen species and nitric oxide than pro-inflammatory cytokines upon stimulation, then into non-classical monocytes, with roles in tissue repair and inflammation resolution and producing TNF-α and IFN type I upon stimulation. This cytokine profile was reflected in the scRNAseq subsets and could explain qualitative differences between the responses to HB-201 and HB-202 infection (Figures 4F,G). Monocytes in HB-202 infected condition predominantly expressed *IL6* as well as *CCL3* and *CCL4*, consistent with a response primarily driven by classical monocytes. In HB-201 infected samples, *TNF* expression but not *IL6*, *IL1A* and *IL1B* were increased together with the IFN type I module described above, consistent with a response driven by non-classical monocytes. Additionally, the expression of *CCL2* and *CCL5*, two chemokines induced by type I IFN, was increased in HB-201. Although the highest inflammatory cytokine levels were detected in monocytes, the observed profiles in cDC1 and cDC2 mirrored those of monocytes, albeit with lower levels.

Together, these findings indicate that whereas the difference between HB-201 and HB-202 in induction of IFN responses was predominantly quantitative, the difference in inflammatory responses is qualitative. HB-201 infection is associated with a higher fraction of non-classical monocytes, accompanied by the production of *TNF*, *CCL2* and *CCL5*. On the other hand, HB-202 has a more subtle effect on monocyte populations and induces a cytokine response characterized by *IL6*, *CCL3* and *CCL4*.

### 2.5 Effect of HB-201 and HB-202 on T cell priming signals

T cell priming and activation requires three signals: 1) antigen presentation by APCs via MHC class I or II, which engages with the T cell receptor (TCR), 2) costimulatory signals provided by costimulatory molecules on APCs with ligands on T cells, such as CD80 and CD86 and 3) a cytokine environment driving T cell proliferation and differentiation. The DC subsets with the strongest T cell priming potential are thought to be cDC1, which specialize in direct MHC I presentation, cross-presentation and induction of CD8^+^ T cell responses, and cDC2, specialized in MHC II presentation for CD4^+^ T cell priming.

Having observed that HB-201 and HB-202 had different infection efficiency of cDC1 and induced a different subset of proinflammatory cytokines, we sought to determine how the overall T cell priming programs were modulated in cDC1 and cDC2 by each vector. We examined the expression of a module for each of the three signals: one Antigen Presentation module including genes associated with antigen processing (*TAP1*, *TAP2*, *PSMB8*, *PSMB9*, *PSMB10*), MHC I presentation (*HLA-A*, *HLA-B*, *HLA-C*, *B2M*, *NLRC5*) and MHC II presentation (*HLA-DRA*, *HLA-DRB1*, *HLA-DPA1*, *HLA-DPB1*, *HLA-DQA1*, *HLA-DQB1*, *CD74*, *CIITA*), one Costimulation module containing costimulatory molecules *CD40*, *CD80*, *CD86*, *CD83*, *CD70*, *TNFSF4*, *TNFSF9* and *ICOSLG*, and one T cell cytokine module including *IL12A*, *IL12B*, *IL23A*, *IL15*, *IL6*, *TNF*, *IL27*, *IL1B*, *IFNA1* and *IFNB1*.

**FIGURE 5:**
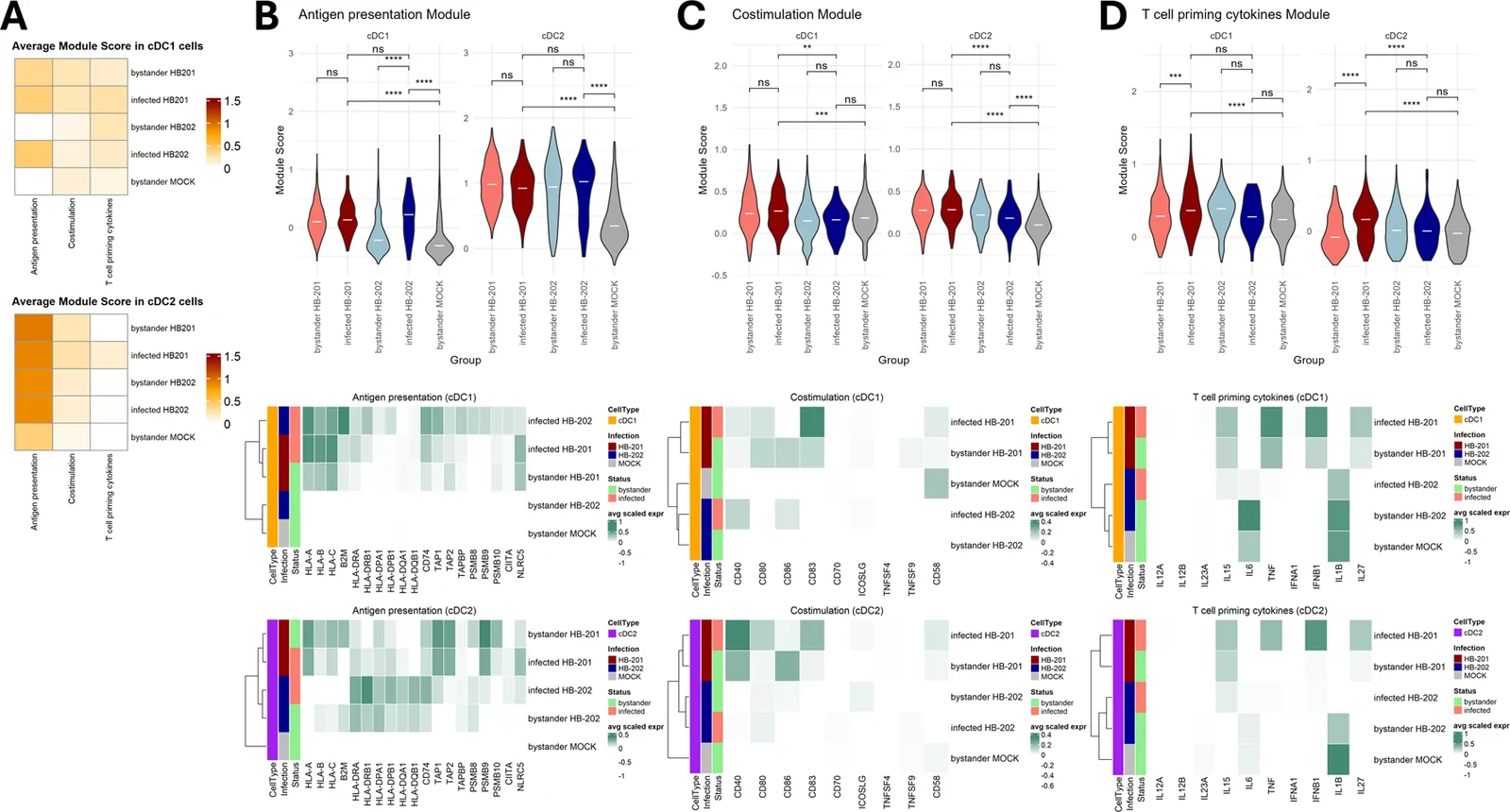
Differential T Cell priming profiles of HB-201 and HB-202: CD8^+^- dominant versus balanced CD4^+^/CD8^+^ responses. **(A)** Expression profiles of Antigen presenting, costimulation and T cell priming cytokines modules across infection conditions and status within one cell type (cDC1 on top, cDC2 on bottom). **(B–D)** Detailed insights on each of the modules separately. Violin plots show significant differences between infection condition and status assessed using Wilcoxon rank-sum test with unadjusted p-values (*p < 0.05, **p < 0.01, ***p < 0.001). Expression plots below show scaled gene expressions of all genes used in the module.

Infection with HB-201 and HB-202 induced a comparable increase in antigen presentation module scores in both cDC1 and cDC2, whereas the increase in costimulation and T cell priming cytokines modules was more pronounced after HB-201 than HB-202 infection in both cell subsets (Figure 5A). Infected cDC1 were enriched primarily in markers associated with antigen processing (*TAP1*, *TAP2*) and MHC I presentation (*HLA-A*, *HLA-B*, *HLA-C*), whereas infected cDC2 upregulated markers associated with MHC I presentation upon HB-201 infection and markers associated with MHC II presentation (*HLA-DRA*, *HLA-DRB1*, *HLA-DPA1*, *HLA-DPB1*, *HLA-DQA1*, *HLA-DQB1*) upon HB-202 infection (Figure 5B). HB-201 infection was accompanied by a higher and broader increase in co-stimulatory molecules than HB-202. HB-201 induced *CD40*, *CD80*, *CD83*, *ICOSLG* and *CD58* in both cDC1 and cDC2, whereas induction was limited to *CD40*, *CD86* and *ICOSLG* in cDC1 and *CD80* and *ICOSLG* in cDC2 for HB-202 (Figure 5C). The induction of cytokines involved in T cell priming, too, was more pronounced upon HB-201 infection. In both cDC1 and cDC2 infected with HB-201, an increase in *IL15*, *TNF*, *IFNB1* and *IL27* was noted, whereas only *IL15* was upregulated by HB-202 (Figure 5D). Furthermore, bystanders cDC1 and cDC2 in HB-202-infected samples contributed to the production of *IL6* and *IL1B*, providing an inflammatory milieu for T cell priming.

Although antigen-specific T cell responses are not expected to occur in this *in vitro* system in the absence of organized lymphoid structures, early activation due to cytokine signaling and co-stimulatory signals can be detected. Consistent with the observations above, we measured T cell activation signatures in both HB-201 and HB-202 infected samples (Supplementary Figure S7). CD4^+^ and CD8^+^ T cells in both infection conditions expressed *CD69*, indicating early T cell activation, and *IRF4*, a transcription factor critical for T cell differentiation. In contrast, CD4^+^ T cells expressed *BATF*, a transcription factor required for Tfh development, only in HB-202 infected samples. Finally, consistent with IFN signaling upon HB-201 infection, CD4^+^ and CD8^+^ T cells in HB-201 infected samples showed higher activation of *STAT1*, a transcription factor downstream of IFN type I.

Taken together, in human DCs HB-201 induced markers associated with MHC I-mediated antigen presentation, with broad induction of costimulatory molecules and type I IFN signaling, a profile generally associated with induction of polyfunctional CD8^+^ T cell responses. HB-202, on the other hand, induced both MHC I and MHC I antigen presentation, more limited costimulatory signals and pro-inflammatory cytokine signals, suggesting a qualitatively distinct, more balanced CD4^+^ and CD8^+^ T cell induction potential.

### 2.6 T cell responses in patients treated with HB-201 and HB-202

To determine whether these hypotheses could be verified in the context of *bona fide* antigen-specific T cell responses, we assessed patients treated with HB-201 or HB-202 as part of the clinical study HB-200-001 (NCT04180215). The Phase 1 cohort described herein comprises 32 patients treated with HB-201 one vector therapy and 47 patients with alternating HB-202/HB-201 two-vector therapy, for which the first dose was HB-202.

**FIGURE 6:**
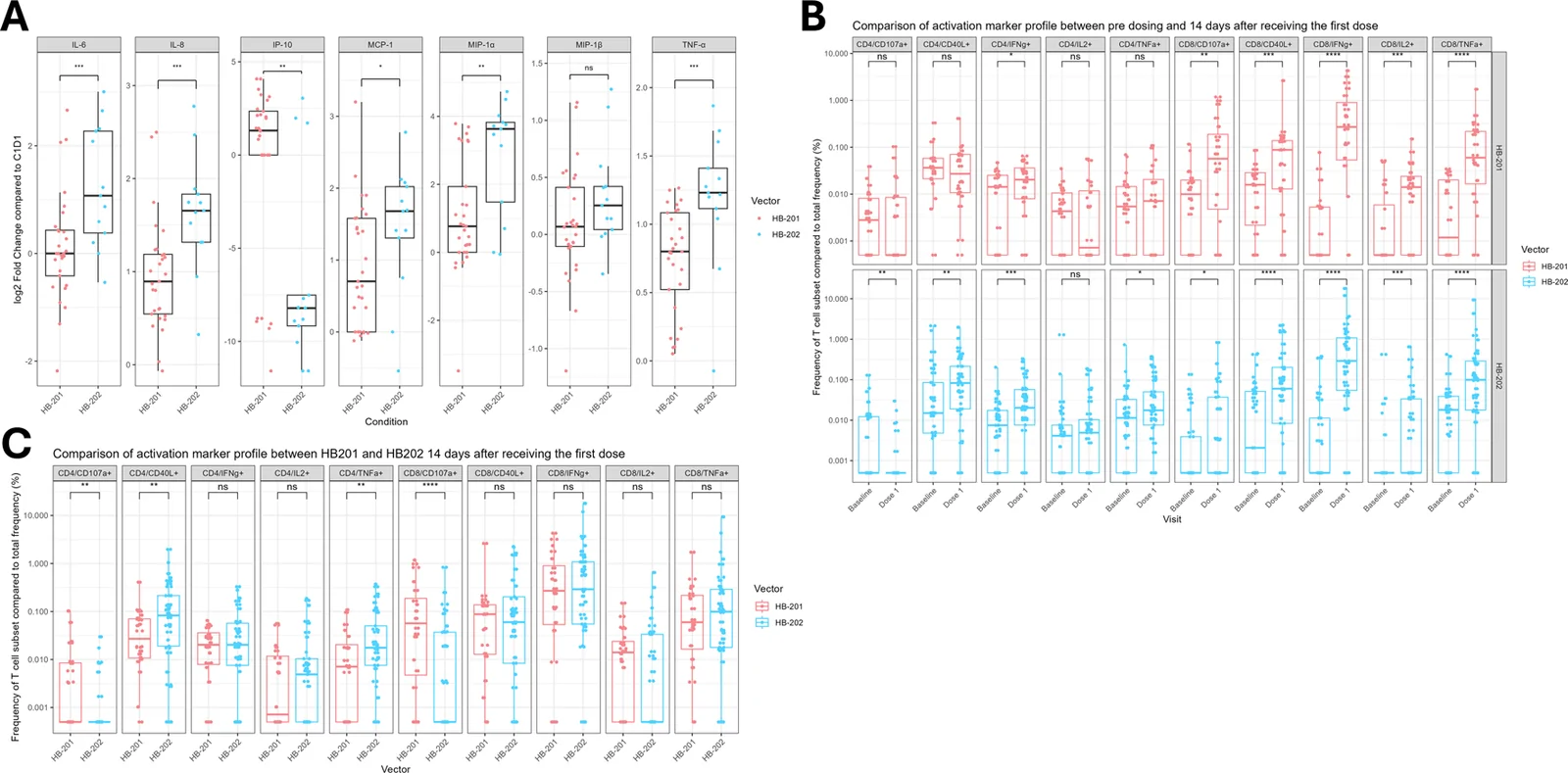
HB-201 and HB-202 induce different polarization of T cell responses in treated patients. **(A)** Cytokine expression of selected markers measured with MSD assay. Y-Axis plots log2 fold change in cytokine concentration from baseline (C1D1) to post-first-dose (C1D4). Data is split by cytokine and infection condition and differences in log2 fold change from baseline (C1D1) between HB-201 and HB-202 were assessed using the Wilcoxon rank-sum test. P-values were adjusted for multiple testing using the Benjamini-Hochberg false discovery rate correction. Adjusted p-values are shown (*p < 0.05, **p < 0.01, ***p < 0.001, ****p < 0.0001). **(B)** ICS staining of T cells isolated from patient PBMCs and stimulated with E7E6 peptide pool. Boxplots are split by marker, T cell subset, treatment type and time point. The y-axis shows the percentage of T cells expressing the specific marker. Significance was assessed using Wilcoxon rank-sum test with unadjusted p-values (*p < 0.05, **p < 0.01, ***p < 0.001, ****p < 0.0001). **(C)** Frequency of T cells expressing the activation markers, directly comparing HB-201 and HB-202 treatment 14 days after first dosing. Significance was assessed using Wilcoxon rank-sum test with unadjusted p-values (*p < 0.05, **p < 0.01, ***p < 0.001, ****p < 0.0001).

To evaluate the innate immune signatures specific to each vector, we profiled serum cytokines 3 days after the first dose of HB-201 or HB-202 (Figure 6A). As observed in the *in vitro* PBMC infection model, HB-201 induced higher IFN responses in patients than HB-202, as evidenced by the induction of *CXCL10*/IP-10 (p = 0.006), an IFN-stimulated gene. Levels of IFN-α and IFN-β were initially measured in a subset of patients, but due to low assay sensitivity the data was not conclusive. HB-202, on the other hand, induced higher levels of pro-inflammatory cytokines IL-6 (p = 0.001), *CCL3*/MIP1α (p = 0.004), TNF-α (p = 0.0007) and *CCL2*/MCP1 (p = 0.043) in patients than HB-201. Together, this data is consistent with the hypothesis that HB-201 triggers an innate immune response dominated by IFN type I signaling in humans, whereas HB-202 triggers a predominantly inflammatory response.

We then looked at the T cell response to the E7E6 transgene encoded in both vectors, in the blood, at baseline and 2 weeks after the first dose. The frequency of CD4^+^ and CD8^+^ T cells responding to E7 or E6 was determined by intracellular cytokine staining for IFN-γ, TNF-α, IL-2, CD40L and CD107α following stimulation of PBMCs with an overlapping E7E6 peptide pool (Figure 6B, upper panel). After one dose of HB-201, we saw a significant increase in E7E6-specific CD4^+^ T cells expressing IFN-γ (median fold change from baseline 1.79-fold, range 0.03–108.4, p < 0.05), but in none of the other cytokines. However, we measured significant increases in E7E6-specific CD8^+^ T cells expressing CD107α (median fold change from baseline 4.87-fold, range 0.02–2364.00, p < 0.01), CD40L (median fold change from baseline 3.41-fold, range 0.10–262.00, p < 0.001), IFN-γ (median fold change from baseline 99.28-fold, range 0.10–8590.40, p < 0.0001), IL-2 (median fold change from baseline 5.08-fold, range 0.03–299.20, p < 0.001) and TNF-α (median fold change from baseline 29.50-fold, range 0.02–3449.20, p < 0.0001), reflecting strong multi-cytokine CD8^+^ T cell responses (Figure 6B, upper panel).

After one dose of HB-202, both multi-functional E7E6-specific CD4^+^ and E7E6-specific CD8^+^ T cell responses were detected. The frequency of E7E6-specific CD4^+^ T cells expressing CD40L (median fold change from baseline 3.83-fold, range 0.00-3938.00, p < 0.01), IFN-γ (median fold change from baseline 3.00-fold, range 0.01-558.40, p < 0.001) and TNF-α (median fold change from baseline 2.84-fold, range 0.00-744.80, p < 0.05) significantly increased (Figure 6B, lower panel). In parallel, E7E6-specific CD8^+^ T cells responses were also detected, as evidenced by the increased frequency of CD8^+^ T cells expressing CD107a (median fold change from baseline 1.00-fold, range 0.004-283.60, p < 0.05), CD40L (median fold change from baseline 3.44-fold, range 0.004-4376.40, p < 0.0001), IFN-γ (median fold change from baseline 92.84-fold, range 0.04-35823.60, p < 0.0001), IL2 (median fold change from baseline 1.00-fold, range 0.001–1298.20, p < 0.001) and TNF-α (median fold change from baseline 5.39-fold, range 0.00–18719.00, p < 0.0001).

Comparing the frequencies of E7E6-specific T cells after one dose of HB-201 of HB-202 showed that although both vectors elicited comparable frequencies of E7E6-specific T cells, their functional profile showed subtle but notable differences (Figure 6C). Both vectors elicited a higher frequency of E7E6-specific CD8^+^ T cells than E7E6-specific CD4^+^ T cells, and there was no significant difference in the magnitude of responses for CD4^+^ and CD8^+^ T cells expressing IFN-γ between both HB-201 and HB-202. However, HB-201 induced a significantly higher frequency of CD8^+^ T cells expressing CD107a, a cytotoxic degranulation marker, and IL-2 than HB-202, indicating a functional polarization towards antiviral cytotoxic T cell responses. On the other hand, HB-202 induced significantly more CD4^+^ T cells expressing CD40L and TNF-α, indicating that HB-202 induces more functional CD4^+^ T cell response compared to HB-201. Taken together, this data is consistent with the hypotheses developed based on the innate immune signatures described above.

## 3 Discussion

Viruses utilized as delivery vectors naturally stimulate innate immune responses to boost the generation of effective T cell responses. Viral infections are known to induce type I IFNs, which regulate the infection and inflammatory responses by attracting immune cells and by facilitating the development of B cell- and T cell-mediated adaptive immune responses. These adaptive immune responses are tightly regulated by a combination of signals that determine both their strength and polarization. In particular, T cell responses are shaped by an interplay of antigen presentation, co-stimulatory signals and cytokines, resulting in different functional profiles. Therefore, it is not unexpected that even minor variations in the interaction between viruses and their host cells can influence downstream T cell immunity.

LCMV and PICV are closely related viruses which were selected as complementary vectors for immunotherapy due to the lack of antigenic cross-reactivity between their backbones. Having two related vectors enables treatment strategies with repeated heterologous prime-boosts, which limit the interference of anti-vector immunity and focuses the immune system towards the antigenic payload. While the biology of LCMV infection is relatively well characterized due to its use as a prototypical strong T cell response inducer, little is known about PICV. Thus, studies of the interaction of the virus with their human host are necessary to understand the contribution of each virus in shaping T cell responses when used as a vector for immunotherapy.

In this study, we developed an *in vitro* human PBMC infection model to characterize the interaction of the two vectors with the human host and to better understand the effect of the viral vector on T cell responses induced. Although this model does not fully recapitulate the potential infection targets and cell-to-cell interactions after intravenous administration in patients, it enables characterization of relevant APCs in a controlled experimental system. We used single-cell RNA sequencing (scRNAseq) to achieve cell type subset-specific profiling of the responses to infection, as different subsets of antigen-presenting cells specialize in the polarization of different downstream immune responses. As models, we selected two recombinant LCMV and PICV vectors carrying the same HPV-16 E7E6 transgene (HB-201 and HB-202). These vectors were previously under clinical investigation for their application in the treatment of head and neck cancer associated with the high-risk, oncogenic human papillomavirus genotype 16 (HPV16). We included both healthy and HPV-16+ head and neck cancer patients as PBMC donors to ensure that clinically-relevant donors were represented. As expected, differences could be observed in the baseline cell composition and transcriptional profiles of the healthy donors and cancer patients. However, these are not described here as the numbers of donors in each category are too small for formal comparisons.

Confirming previous data, we could show that HB-201 and HB-202 had a strong tropism for antigen-presenting cells (APCs) (Raguz et al., 2024). Monocytes, conventional dendritic cells (cDC1 and cDC2) and plasmacytoid dendritic cells (pDC) were efficiently infected by HB-201 and HB-202. The highest levels of genomic RNA per infected cell were detected in monocytes and cDC1, followed by cDC2 and pDCs. Interestingly, HB-202 exhibited a lower level of infectivity in cDC1 cell type. This finding is in contrast to the results reported by Raguz et al. (2024) where infection rates for cDC1 were comparable for both viral vector backbones. A plausible explanation for this difference is that a higher viral dose per cell was used and a spinoculation step was included in the previous study, potentially overcoming the barrier to infection we observed. Furthermore, in our study infection was defined by the presence of viral RNA in the cell, which does not discriminate between productive and abortive infection, whereas in Raguz et al. (2024) transgene expression was assessed. Nonetheless, infection in a human host after i. v. Injection, as administered in clinical trials of arenavirus vectors, is likely to achieve a relatively low multiplicity of infection closer to our experimental system.

HB-201 induced strong type I interferon-related responses, whereas HB-202 triggered a comparatively weaker activation with no to minimal expression of *IFNB1*. This was true in all infected subsets. This pattern aligns with findings reported in other studies measuring IFN type I in cells infected with wild-type LCMV and wild-type PICV, showing minimal IFN-*β* expression in PICV wild-type infections whereas LCMV infections trigger high levels of IFN-*β* production (Pythoud et al., 2012; Huang et al., 2015). In turn, levels of IFN-stimulated genes, which represent downstream effectors of IFN activation with antiviral activity, were dramatically higher in HB-201-infected and bystanders than in HB-202-infected cells. This suggests that both viruses have evolved distinct mechanisms to engage, and evade restriction by the IFN system. Based on our data, it is likely that PICV acts at a very early stage of the response, by either passively “hiding” molecular patterns acting as triggers for PRRs like RIG-I or TLR3, or actively antagonizing early signaling events leading to the production of IFN type I. On the other hand, PRRs can induce IFN type I production more efficiently in response to LCMV infection, which is likely to antagonize its antiviral effects downstream of ISG induction. Indeed, us and others have shown that once infection is established, LCMV is resistant to inhibition by exogenously added IFN type I (Moskophidis et al., 1994; Ou et al., 2001).

Additionally, inflammatory responses to the two viral vector backbones were markedly different. Monocytes were identified as the primary contributors to the inflammatory response in both HB-201 and HB-202-infected samples. In the HB-201 infection condition, an increase in the proportion of non-classical monocytes was observed, accompanied by upregulation of *TNF*, *CCL2*, *CCL5*, *CASP1* and *GSDMD*. In contrast, the HB-202 infection condition was associated with less effect on monocyte subsets and an increased expression of *IL1B*, *IL1A*, *IL6*, *CCL3* and *CCL4*. This finding aligns with a previously published study demonstrating that non-classical monocytes respond to stimuli with lower expression of *IL1B* and *IL6*, promoting resolution of inflammation (Boyette et al., 2017). Interestingly, Lukashevich et al. observed an LCMV strain dependent innate immune activation. In that study, the LCMV-WE strain, utilized as genomic backbone for the HB-201 construct, also did not induce *IL6* expression upon infection (Johnson et al., 2024).

To assess T cell priming, antigen presentation and costimulation, we examined gene expression profiles in cDC1 and cDC2 dendritic cell subsets. While cDC1s are the most prominent cross-presenters when antigens are acquired exogenously, cDC2s also possess the capacity to prime cytotoxic CD8^+^ T cells, especially upon IFN type I stimulation and when the antigens are expressed intracellularly (Hamouda and Laoui, 2025). In HB-201-infected cDC1 and cDC2, intracellular antigen expression was accompanied by upregulation of the MHC class I processing and presentation machinery, upregulation of T cell costimulatory molecules CD40, CD80 and CD83, and a cytokine environment dominated by IL15, IFNB1, TNF and IL27. Together, this suggests strong CD8^+^ T cell priming potential and cytotoxic polarization. On the other hand, HB-202-infected more efficiently cDC2, and induced in these cells MHC class II processing and presentation machinery, together with an inflammatory cytokine milieu. This is expected to induce a more balanced CD4^+^ and CD8^+^ T cell response.

To confirm these hypotheses in a clinically-relevant setting, we analysed patient T cell activation and functionality following HB-201 and HB-202 treatment. After one dose of HB-201, strong CXCL10 responses, indicative of IFN type I responses were detected in the serum of patients. T cell responses to the E7E6 transgene were restricted to the CD8^+^ subset and expressed the highest levels of the cytotoxic degranulation marker CD107a. On the other hand, patients treated with one dose of HB-202 exhibited higher serum signatures of inflammatory responses, and CD4^+^ T cell responses to E7E6 could be detected together with CD8^+^ T cell responses with a lower cytotoxic profile.

While antitumor immunity has traditionally been attributed to cytotoxic CD8^+^ T cells, CD4^+^ T cells can also contribute substantially by secreting cytokines and chemokines that enhance CD8^+^ T-cell function, recruit and activate other immune cells, promote immune memory, and potentially reduce immune escape, although the relative importance of these mechanisms varies across patients and tumor microenvironments. In the absence of a clear correlation of antitumor immunity, the impact of the different T cell responses induced by HB-201 and HB-202 on patients’ clinical outcomes remains to be determined.

In conclusion, this study highlights that although closely related, LCMV and PICV elicit distinct differences in innate and adaptive immune responses when used as viral vector backbones. During early stages of infection, each virus generates a unique innate signaling environment, which is associated with qualitative differences in the T cell response to the encoded transgenes. The described observations provide initial insights into the specific immune responses to PICV and LCMV across innate and adaptive compartments and lay the foundation for future studies exploring how early APC responses influence downstream T cell polarization in arenaviral vector-based therapies. This mechanistic understanding could guide the optimization of treatment regimens tailored to individual disease contexts.

## 4 Methods

### 4.1 Vector generation

HB-201 (artLCMV-E7E6) and HB-202 (artPICV-E7E6) vectors were generated as previously described (Raguz et al., 2024; Schmidt et al., 2020). The vector-encoded antigen (fusion protein of E7 and E6: GenBank accession #K02718 with five mutations to abrogate oncogenic potential 22) coding sequence for artLCMV-E7E6 was synthesized by GenScript and inserted into 2 plasmids encoding the S-Segment of LCMV (clone 13). S-Segment #1 encoded LCMV NP, and S-Segment #2 encoded LCMV GP (LCMV strain WE). For the generation of artPICV-E7E6, the identical HPV16-derived antigen was inserted into plasmids encoding the S-Segment of PICV (strain p18), with S-Segment #1 encoding PICV-NP and S-Segment #2 encoding PICV-GP. Vectors were generated by transient transfection of BHK21 cells stably expressing LCMV-GP23 and amplified on HEK-293 cells. The replication competent viral particles titer was determined by focus forming assay.

### 4.2 Donor and patient samples

PBMCs from patients were obtained from patients enrolled in a clinical trial (ClinicalTrials.gov ID:NCT04180215). This study was a first-in-human Phase I/II, multinational, multicenter, open-label study enrolling patients with metastatic/recurrent HPV-16^+^ HNSCC. In the Phase I dose escalation, patients were treated intravenously with HB-201 alone or with HB-202/HB-201 alternating two-vector therapy. In the Phase II dose expansion, HB-201 or HB-202/HB-201 at the selected dose were given as monotherapy or in combination with pembrolizumab. Vectors were administered for three cycles at 21-days interval, then at 42-days interval for the remaining treatment time. The primary objective was safety, secondary objective was preliminary antitumor activity, and exploratory objectives included analysis of the immune response to treatment.

Buffy coats from healthy donors were purchased from the Red Cross blood donation service. The samples were diluted in phosphate-buffered saline (PBS) and layered over Ficoll Paque Plus (Cytiva) prior to density gradient centrifugation at 800xg for 20 min at room temperature with no brake. The interphase containing the PBMCs was carefully collected, washed with PBS and pelleted by centrifugation (300xg for 10 min), then cryopreserved with Cryostore CS10 (Stemcell) until further use.

### 4.3 Infection of human PBMC and enrichment for sequencing

Human peripheral blood mononuclear cells were thawed, seeded with a concentration of 1 million cells per well in a 24-well plate in complete medium (RPMI 1640 medium (Gibco) + 10% human serum (Sigma-Aldrich), 1% GlutaMax, 1% Sodium Pyruvate, 1% NEAA, 1% PenStrep, 0.01% b-Mercaptoethanol) and infected at a MOI of 1. The cells were incubated for 5–6 h at 37 °C before replacing the cell culture supernatant with fresh medium Infection was carried out for a total of 24 h.

After 24 h, the cells were harvested using 0.05% Trypsin-EDTA, transferred in FACS buffer (PBS, 2.5% FBS, 10 mM EDTA), blocked (human Fc Block, BD Biosciences) and stained with PE anti-human HLA-DR Antibody (BioLegend). After 30 min at 4 °C, the cells were washed once with FACS buffer and once with MACS buffer (MACS BSA stock solution 1:20 with autoMacs Rinsing Solution from Miltenyi) before adding 20 µL Anti-PE MicroBeads for enrichment of HLA-DR expressing cells by magnetic separation.

Enrichment efficiency was checked for two donors by flow cytometry. PBMCs were resuspended in FACS buffer (PBS, 2.5% FBS, 10 mM EDTA), blocked (human Fc Block, BD Biosciences) and stained with PE anti-human HLA-DR (BioLegend), BUV395 anti-human CD3 (BD), FITC anti-human CD4 (Biolegend), BUV737 anti-human CD16 (BD), APC anti-human CD11b (Biolegend), BV785 anti-human CD19 (Biolegend), BV421 anti-human CD123 (Biolegend), BV650 anti-human CD33 (Biolegend), PE-Dazzle 594 anti-human CD11c (Biolegend), AF700 anti-human CD14 (Biolegend), APC-Cy7 anti-human CD1c (Biolegend) and PE-Cy7 anti-human CD141 (Biolegend) antibodies. After 30 min at 4 °C, the cells were washed twice with FACS buffer. Samples were acquired on a BD LSRII flow cytometer and analysis was performed using FlowJo v10 as outlined in Supplementary Figure S3.

### 4.4 Library preparation and sequencing

Sequencing libraries were prepared following the manufacturer’s protocol for single-cell RNA sequencing using the 10x Genomics Chromium GEM-X Single Cell 5′ Immune Profiling kit. Quality and quantity of single final libraries were determined using the Qubit (Thermo Fisher) and the Tapestation 4200 system (Agilent). Final quality control and sequencing were performed by CeGat GmbH (Tübingen, Germany). Samples were sequenced in one run with an Illumina NovaSeq 6000 system (S2 100-cycle cartridge) with calculated requirements of at least 30,000 reads per cell and 4,000 cells per sample. Data was delivered as raw, untrimmed FASTQ files.

### 4.5 Single cell RNA sequencing data analysis

FASTQ files were processed with *CellRanger v8.0.0 (10x Genomics)* for demultiplexing and alignment to the hg38 reference genome including the sequences of the viral coding regions of HB-201 and HB-202. Generated expression matrices were analysed in *R v4.1.2* with *Seurat v5.1.0* (Hao et al., 2024). Quality control excluded cells with <100 or > 8,000 detected genes, > 50,000 reads or > 15% mitochondrial transcripts. Data was log-normalized, scaled, centered and reduced by principal component analysis (PCA). Clusters were generated by constructing K-nearest neighbor (KNN) graphs followed by clustering with the Leiden algorithm and a resolution of 1.2. Cell types were annotated based on several marker genes for respective cell subtypes. Genes were grouped to modules for comparison of immune signatures and visualization of differences. Visualization of gene expression was performed using *DimPlot*, *DotPlot*, *RidgePlot* and *FeaturePlot* from the *Seurat* (Hao et al., 2024) package. Violin plots, bar plots and box plots were generated with *ggplot2*, and heatmaps were generated with *pheatmap* (Kolde, 2024) and *ComplexHeatmap* (Gu, 2022; Gu et al., 2016).

*Uniquely upregulated genes in cell clusters and groups were identified with FindMarkers function from the Seurat* (Hao et al., 2024) package and visualized with *VennDiagram* (Chen and Boutros, 2011) package.

No correction for batch effect was performed as all samples were prepared in individual libraries and sequenced in a single run.

### 4.6 Serum cytokine analysis

Cytokine concentrations in patient serum samples were quantified using a U-PLEX multiplex immunoassay (Meso Scale Discovery, MSD®) comprising 30 analytes, following the manufacturer’s instructions. Briefly, serum samples and standards were added to pre-coated U-PLEX plates containing analyte-specific capture antibodies, followed by incubation, washing, and detection with SULFO-TAG–labeled secondary antibodies. Plates were read using an MSD electrochemiluminescence (ECL) detection system, and analyte concentrations were determined from standard curves generated using the associated MSD software. Raw data were exported as an Excel spreadsheet and imported into R (v4.1.2) for data curation, and visualization.

### 4.7 Intracellular staining assay

Briefly, cryopreserved patient PBMC were thawed, counted with the EasyCyte™ HT system, distributed into a 96-well plate at 0.5 × 10^6^ cells/well and stimulated for 6 h in presence of HPV-16 E6E7peptide pool of 15-mers with 11 amino acids overlap and > 90% purity at 2.0 mg/mL/peptide in the presence of the APC-conjugated anti-CD107a antibody. Medium with matched DMSO concentration served as non-stimulated (NS) control. As a cell functionality control, cells from each sample analyzed were also plated at 2 × 10^5^ cells/well in presence of SEB at 0.25 mg/mL. Protein transport inhibitors (Golgi Plug and Golgi Stop) were added to the culture after 1 h. Following stimulation, PBMC were washed, non-specific antibody binding was blocked using an Fc Block solution for 10 min at Room Temperature (RT), and PBMC were stained with surface marker antibodies and with a viability dye for 20 min at 2 °C–8 °C. Cells were then washed, fixed and permeabilized in BD Cytofix/Cytoperm™ buffer for 20 min at 2 °C–8 °C. Cells were washed with BD PermWash™, and stained with the intracellular antibody cocktail for 20 min at 2 °C–8 °C. Then, cells were washed with BD PermWash™ and re-suspended in 2% FBS prior to sample acquisition on a BD cytometer.

In each ICS run, an inter-run control sample was analyzed along with the clinical samples to monitor assay performance. The control PBMC sample from a HCMVpp65-responsive healthy donor was stimulated with medium/DMSO, HCMVpp65 peptide pool or SEB in single replicates.

ICS data were analyzed following a standardized gating strategy. Briefly, events were first gated to exclude cell debris based on forward and side scatter (FSC/SSC) parameters and removing non-viable cells using the Live/Dead Fixable Near-IR viability dye. The remaining viable lymphocyte population was gated on CD3^+^ T cells, which were subsequently subdivided into CD4^+^ and CD8^+^ subsets. Within each subset, cells were further analyzed for expression of individual functional markers, including IL-2, TNF-α, IFN-γ, CD40L, and CD107a. Boolean gating was applied to generate combinations of cytokine-positive and activation marker–positive populations within the CD4^+^ and CD8^+^ compartments. Processed data were exported for data cleaning and plotting in R. For each sample, background signal was corrected by subtracting the frequency of each cytokine-positive or activation marker–positive population observed in the unstimulated control condition from the corresponding stimulated condition. To avoid artificially low or zero values after background subtraction, any resulting frequencies below 0.001 were replaced with a value of 0.0005. This threshold was defined as an approximate limit of detection (LOD), based on the criterion that a minimum of 10 positive events are required for reliable detection. Given an average input of approximately 200,000 total lymphocytes per sample, of which roughly 10% (≈20,000) represent CD4+/CD8^+^ T cells, this corresponds to a frequency threshold of 0.0005 for confident positivity.

The data was imported in *R* (v4.1.2) and plotted with the *ggplot2* library.

### 4.8 Direct RNA sequencing of *in vitro* transcribed RNA

*In vitro* transcribed (IVT) RNA was prepared with a linearized template DNA originating from a plasmid encoding for the S_GP_ segment. The template DNA was linearized by PCR amplification with primers aligning to the first bases of the 5′UTR and the last bases of the 3′UTR, ensuring amplification of the full viral genome segment. The forward primer aligning to the 5′UTR consisted of the region aligning to the viral genome and a T7 overhang, ensuring T7 priming during *in vitro* transcription. IVT RNA was synthesized using the T7 RiboMAX kit (Cat.: P1320) according to manufacturers protocol incubating at 37 °C for 30 min before incubating with RQ1 RNase-Free DNase (Cat.: M6061) at 30 °C for 30 min. The IVT RNA was purified with the RNeasy MinElute Cleanup Kit (Cat.: 74204) following the manufacturer’s protocol, afterwards the product was quantified with the Qubit RNA BR assay and RNA integrity and size distribution was verified using the Agilent TapeStation 4500. For the polyadenylated testing condition the purified IVT RNA was polyadenylated using the NEB Poly(A) Polymerase Tailing Kit (New England Biolabs, Cat. M0276) with an incubation time of 30 min at 30 °C. The polyadenylated IVT RNA was purified and cleaned up with RNAClean XP beads (Beckman Coulter, Cat: A63987). Quantified RNA served as input to Oxford Nanopore direct RNA Sequencing Kit (SQK-RNA002), the protocol was executed with minor adaptations to the manufacturers recommendations. After adjusting the RNA concentration to 500 ng in 9 μL, the sample was linearized by incubating at 70 °C for 2 min before placing it on ice. In the next step, the adapters were ligated to the RNA using T4 DNA ligase. Here either the provided RT Adapter or a custom oligo adapter targeting the viral 3′UTR (prepared following ONT provided protocol) were used. Deviating from the manufacturers recommendations, the reverse transcription of the RNA was skipped and the sequencing adapters were added to the single stranded RNA before sequencing on a GridION sequencer using a R9.4.1 flowcell (FLO-MIN106) for 40 h.

The data was aligned to the viral reference genome with *minimap2* and the *-ax map-ont* parameter, followed by using *bam-readcount* tool for coverage evaluation across the reference and plotting in *R* (v4.1.2) with the *ggplot2* library.

### 4.9 Statistics

Statistical comparisons between groups were performed using the Wilcoxon rank-sum test implemented in the *ggpubr* package in R using the *stat_compare_means* function. This non-parametric test was used to compare distributions between two groups, specified in the comparisons parameter. Unadjusted p-values were used for all comparisons, except for the cytokine measurements in Figure 6A. Here p-values were adjusted for multiple testing using the Benjamini-Hochberg false discovery rate correction. Two-sided tests were performed without assuming normal distribution of the data. Statistical significance was annotated on the plots using conventional thresholds and standard notation (*p < 0.05, **p < 0.01, ***p < 0.001).

### 4.10 Ethics approval

Patient samples are obtained from HOOKIPA Pharma’s phase I/II trial NCT04180215, where the vectors HB-201: artLCMV-E7E6 and HB-202: artPICV-E7E6 were evaluated alone or in combination with pembrolizumab in metastatic or recurrent HPV16+ HNSCC patients.

The study protocol and all amendments were reviewed and approved by an Independent Ethics Committee (IEC) or Institutional Review Board (IRB) prior to initiation. The study was conducted in accordance with the principles of the Declaration of Helsinki, the International Council for Harmonisation (ICH) Good Clinical Practice (E6 R2) guidelines, and all applicable regulatory requirements. All participants or their legally authorized representatives provided written informed consent after receiving a detailed explanation of the study objectives, procedures, and potential risks. Re-consent was obtained when updated consent forms became available. The use of biomarker samples for research purposes was permitted as specified in the informed consent documentation.

## Data Availability

The analysis code supporting the conclusions of this study is publicly available on GitHub at: https://github.com/sarah37s/Manuscript_scRNAseq_HOOK. The sequencing data have been deposited in the Gene Expression Omnibus (GEO) under accession number GSE338107.
