# Supplementary material for: Innate immune activation profiles are associated with polarization of the T cell response to recombinant arenaviral vectors
Source: Front Syst Biol. 2026 Sep 3;6:1897043. doi: 10.3389/fsysb.2026.1897043 (PMC13581738; doi:10.3389/fsysb.2026.1897043)
Supplement: Supplementary file 1 [file DataSheet1.docx]

**Supplementary file**

**S1 Fig**

**S1 Fig. Coverage distribution of polyadenylated and non-polyadenylated *in vitro* transcribed RNA.** *In vitro* transcribed RNA was sequenced directly without amplification using Oxford Nanopore Technologies (ONT) direct RNA sequencing under three conditions non-polyadenylated RNA with Oligo(dT) sequencing adapters (dark green), polyadenylated RNA with Oligo(dT) sequencing adapters (light green) and non-polyadenylated RNA with sequence-specific primers used as sequencing adapters (orange). The x-axis represents the viral reference sequence with annotation of the viral segment and coverage depth on the y-axis.

**S2 Fig**

**S2 Fig. Optimization of infection conditions for two arenavirus-based vectors.** PBMCs from two healthy donors (HOOK53, HOOK54) were infected with either artLCMV-GFP or artPICV-GFP arenaviral vectors using multiplicities of infection (MOI) 0.2, 1 and 5 and harvested 24 hours, 48 hours and 72 hours after infection. A not infected (MOCK) control was included for identification of background staining. Transduction efficiency was measured by flow cytometry as the percentage of GFP-positive cells.

**S3 Fig**

**S3 Fig. Efficiency of HLA-DR enrichment.** The proportion of APC subtypes in two donor PBMC before and after HLA-DR enrichment step was characterized by flow cytometry. **(A)** Gating strategy. B cells were defined as CD19+, T cells as CD3+, Monocytes as CD3- CD19- CD33+ CD11c+ CD14+, cDC1 as CD3- CD19- CD33+ CD14- CD16- HLA-DR+ CD11c+ CD1c-, cDC2 as CD3- CD19- CD33+ CD11c+ CD14+, cDC1 as CD3- CD19- CD33+ CD14- CD16- HLA-DR+ CD11c+ CD1c+ and pDC as CD3- CD19- CD33+ CD11c+ CD14+, cDC1 as CD3- CD19- CD33- CD11c- CD11b- HLA-DR+ CD123+. **(B)** Quantification of cell types in PBMCs of two healthy donors before enrichment, after HLA-DR enrichment and in the flow-through of HLA-DR enrichment.

**S4 Fig**

**S4 Fig. Quality control filtering and viral gene expression across scRNAseq samples. (A)** Number of cells per sample before and after quality control filtering excluding cells with <100 or >8,000 detected genes, >50,000 reads, or >15% mitochondrial transcripts. **(B)** Viral gene expression detected in each sample based on scRNAseq reads mapping to viral genes. For both panels, healthy donors are labeled HOOK26, HOOK60 and HOOK62, whereas all other labels indicate clinical samples. The timepoints C1D1 (Cycle 1 Day 1) and SCR (Screening) represent baseline measurements from clinical samples.

**S5 Fig**

**S5 Fig. Clustree analysis for selection of clustering resolution.** Clusters were calculated across increasing resolution parameters and visualized using the *clustree*[*[28]*](https://sciwheel.com/work/citation?ids=5672028&pre=&suf=&sa=0) tool. Nodes represent clusters at each resolution and edges indicate how cells transition between clusters as resolution increases. The analysis was used to identify a stable clustering structure and resolution 1.2 was selected for downstream analysis, (highlighted in blue, number 6 in the clusters_4_a legend).

**S6 Fig**

**S6 Fig. Infection rates and viral load across monocyte subsets.** **(A)** Percentage of infected cells in each monocyte subset (classical, intermediate, non-classical) determined by detection of viral gene expression in each single cell. **(B)** The plot shows the normalized number of viral genome copies per monocyte subset corresponding to the infection rates shown above.

**S7 Fig**

**S7 Fig. scRNAseq analysis of T cells shows early T cell priming in HB-201 and HB-202. (A)** UMAP plots showing infection conditions and **(B)** UMAP visualization of identified T cell clusters. **(C)**  Expression of cell type markers (CD3D, CD8A, CD4, NKG7) on the UMAP. **(D)** Violin plots of the same markers used to assign clusters 1, 2, 3 and 4 as CD4+ T cells and cluster 5 and 6 as CD8+ T cells. **(E)** Expression of a panel of activation markers to identify early T cell activation.
